# Stress-Induced Auto-Cannibalism in Patients With a History of Moderate Traumatic Brain Injury

**DOI:** 10.7759/cureus.41232

**Published:** 2023-06-30

**Authors:** Gregory Fenati, Santana Youssoffi, Dustin Phan, Katharine McManus, Fanglong Dong, Michael M Neeki

**Affiliations:** 1 Emergency Medicine, Arrowhead Regional Medical Center, Colton, USA; 2 Emergency Medicine, California University of Science and Medicine, Colton, USA

**Keywords:** emergency department, self-injury, traumatic brain injury, self-cannibalism, auto-cannibalism

## Abstract

A traumatic brain injury (TBI) is a significant factor in injury-related deaths in the United States and may lead to complex psychological disorders. Auto-cannibalism as a sequela of a TBI has yet to be reported in the literature. The current literature regarding such behavior is often associated with psychosis, intellectual disability, or substance use.

A 35-year-old male had a past medical history significant for a TBI a decade ago. He was transferred to the emergency department due to a self-inflicted wound. The patient had been scratching his arms and legs for the last few months and displayed an intense new pattern of self-destructive behavior in the past week. He went through surgical wound debridement and psychiatric evaluation before he was discharged home.

This case depicts the importance of regular, long-term psychiatric, and neurological follow-up for patients sustaining TBIs, regardless of whether or not they were previously deemed stable. A greater understanding of many factors leading to self-destructive behavior following TBIs is needed to improve patient outcomes.

## Introduction

Traumatic brain injuries (TBIs) accounted for 10% of all injuries seen in emergency department (ED) visits and 18.1% of all hospitalizations in 2013 [1]. TBIs also contributed to 29.9% of all injury-related deaths in the United States in 2013 [1]. TBIs may also lead to complex behavioral changes such as increased irritability, aggression, apathy, and impulsivity as well as psychiatric disorders including mood and anxiety disorders, substance abuse, and psychotic disorders [2,3].

TBIs can be classified as mild, moderate, or severe depending on the structural imaging and patient’s Glasgow coma scale, as well as the duration of loss of consciousness, alteration of mental status, and post-traumatic amnesia [4]. They can also be classified by the mechanism of injury as closed head, penetrating, or explosive blast [2,5]. The closed-head TBI is the most common and typically caused by motor vehicle accidents, falls, and sports [2,5]. It ultimately disrupts normal brain function through contact forces, which immediately damage the brain vasculature and neuronal cells underneath the impact site [5]. Additionally, brain displacement due to vibrations and shocks from the impact can compress brain tissue and reduce cerebellar flow [5].

Auto-cannibalism, also known as self-cannibalism, or autosarcophagy, is an act characterized by the consumption of one's flesh. The most frequent cases of auto-cannibalism are reported in patients with moderate to severe brain developmental disability in which there is a global deficiency in cognitive, motor, and social functions [6,7]. The infrequency of documentation in the medical field regarding auto-cannibalism makes it a tenuous diagnosis.

More broadly, self-injurious behavior (SIB) was defined as non-accidental behavior resulting in demonstrable, self-inflicted physical injury without the intent of suicide or sexual arousal. Typically, the behavior is repetitive and persistent [8]. Huisman et al. reviewed primarily SIB in the context of genetic diseases such as Angelman syndrome and Lesch-Nyhan syndrome, all of which are associated with intellectual disability [8]. McClintock and his colleagues also noted a significant association between severe intellectual disability and the prevalence of self-injury [9]. Studies reported that self-injury behavior may range from 4% to 23% in patients with intellectual disability [10-12]. Furthermore, the prevalence and severity of self-injurious behavior increase with age and may persist for decades [13,14].

Such circumstances of deleterious self-injury leading to auto-cannibalism in the literature are scarce. To date, no case of auto-cannibalism after TBIs has been reported in the literature. As such, the evaluation of cases of auto-cannibalism can be challenging in the ED setting. Physicians must consider beyond the observed physical injuries and venture into the psychological and neurological origin of self-destructive behavior. This case adds to the scant literature regarding complications arising from the psychiatric disorder associated with moderate to severe TBIs.

## Case presentation

A 35-year-old male with a past medical history significant for a TBI a decade ago resulting from a motorcycle accident was brought into the ED by emergency medical services from his home due to a self-inflicted wound. He lives with his family and needs help with daily care and supervision. According to the patient’s mother who accompanied him to the ED, the patient had been scratching his arms and legs for the last few months and displayed an intense new pattern of self-destructive behavior in the past week.

He began chewing and biting his arm over the past three days. The patient’s family attempted to stem the biting by wrapping the area in gauze. However, the patient continued to chew through the dressings. In addition, it was stated that his self-injurious behavior manifested predominantly during sleeping hours and times of increased anxiety or stress. His previous psychiatric history was also significant for several episodes of psychiatric hospitalizations in the past few years stemming from suicidal ideation. Furthermore, the family had relayed that he had been taking escitalopram, 10 milligrams, for the past five years for major depression, anxiety, and suicidal ideations.

On physical examination, the patient was disheveled and bedbound and had visible contractures of his upper extremities. He had a blood pressure of 122/93 millimeters of mercury (mmHg), a heart rate of 86 beats per minute, a respiratory rate of 18 breaths per minute, and oxygen saturation of 100% on the room air. The physical exam showed an alert patient. Cardiac auscultation revealed a normal rate and rhythm without murmurs, rubs, or gallops. Bilateral radial and dorsalis pedis pulses were detected at 2+. Respiratory auscultation demonstrated even breath sounds bilaterally. The abdomen was soft, non-tender, and non-distended, with bowel sounds present.

He was wrapped in a blanket from home with his face covered in dried blood. Dehydrated pieces of human flesh and tissue were found among his bedclothes and on his body. Given the large amounts of human flesh notable on different parts of his body and skin surfaces, it was difficult to locate the exact source. During the initial evaluation, the patient expressed some cognitive deficits which were at baseline since his TBI and confirmed by his mother. He was, however, able to accurately state his name and current age. He was also able to recall the cause of his TBI. During the assessment, he denied any suicidal ideations or an intention to harm himself, stating that chewing himself “was calming.”

**Figure 1:**
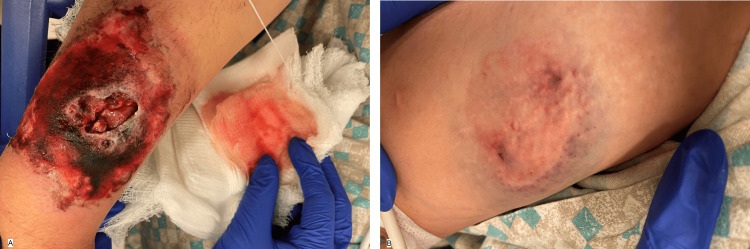
Self-inflicted injury resulting in 12 centimeters x 7.5 centimeters ulcerated wound on the right forearm (A) and a superficial bite wound to the right thigh (B).

Detailed examination after the clean-up of his extremities revealed a well-demarcated, 12-centimeters-long by 7.5-centimeters-wide ulcerated wound on the central area of his right forearm. The wound appeared deep with penetration into muscle layers (Figure 1A). An additional superficial bite wound to his right upper bicep and right thigh was noted (Figure 1B).

His care at the ED included local wound cleansing and intravenous antibiotic therapy with three grams of ampicillin/sulbactam to prevent infection from the contaminated wounds with oral flora. Due to concerns of continued self-harm, the patient was placed on a 48-hour psychiatric hold along with formal psychiatry consultation. In addition, the orthopedic service was consulted to evaluate the need for debridement of the wounds. He was admitted to the hospital for further evaluations and treatment by various specialty services.

During the psychiatric evaluation, the patient continued to deny any suicidal ideation. No delusions or hallucinations were noted, and the patient denied receiving previous psychiatric care. Furthermore, he could not explain how his self-injurious behavior began. After the initial psychiatric evaluation, it was determined that the patient did not fulfill the criteria for an involuntary psychiatric hold. Since the patient reported a correlation between his self-harming behavior and anxiety and stress levels, early intervention with divalproex sodium was deemed beneficial by the psychiatric team. Subsequently, the patient was taken to the operating room the day after admission for wound debridement and cleansing. He continued to be on ampicillin/sulbactam three grams intravenous four times daily. Upon arrival back to the inpatient unit, he attempted to chew through the dressings covering his wounds. Throughout the hospital course, he required frequent sedation and recurrent wound care and ultimately underwent a second surgical wound debridement procedure. The second surgery included a split-thickness skin graft and wound vacuum placement.

The patient finished the rest of his hospital stay with continued antibiotic therapy and was discharged home after he was cleared by the psychiatric team. The adult protective services deemed his family capable of providing care as he is dependent on them to perform his activities of daily living. During the patient’s follow-up appointment, the orthopedic team noted mild bleeding with edema on the lateral dorsal aspect of his wrist, with erythema and fibrotic material present. He was neurovascularly intact and denied any pain. On a follow-up five months after the hospital stay, the patient's mother stated that he continued to bite himself; however, his current wounds are minor.

## Discussion

Most of the literature involving auto-cannibalism is limited to case reports. A handful of instances have been recorded over time, always involving psychosis, intellectual disability, or substance use. Yilmaz et al. described a case of a male prisoner who cut out a piece of his thigh and ate it [15]. The patient was diagnosed with psychosis and the study suggested that self-harm behavior manifests when an individual is unable to express aggression. Another case report described a patient diagnosed with obstructive sleep apnea who would chew on his hands during sleep [16]. The patient was treated successfully with continuous positive airway pressure and his self-injurious behavior ceased. An association between auto-cannibalism and major-depressive disorder, anxiety disorder, or TBI has yet to be reported in the literature.

Primary brain injuries encompass the immediate impact of different mechanical insults. In contrast, secondary brain injuries include events that progress into delayed and prolonged secondary damages, lasting from hours to years. Ng and Lee identified the factors leading to secondary brain injuries as glutamate receptor excitotoxicity, mitochondrial dysfunction, oxidative stress, lipid peroxidation, neuroinflammation, axon degeneration, and apoptotic cell death [5]. Many of these factors, particularly reactive oxygen species, are likely to be key mechanisms to link TBI and increased risk of neurodegeneration [17]. A study by Millar et al. assessed a cohort of 396 patients after a head injury 15 to 25 years later [18]. They reported that roughly two-thirds of the patients had deteriorated between six months and follow-up, as measured by their Glasgow outcome scale. The psychiatric episodes with suicidal ideations and reported anxiety of our patient have been progressively worsening. This may lead us to consider progressive neurodegeneration as a secondary injury following the TBI 10 years ago; however, it is unclear without proper neuroimaging such as computed tomography, magnetic resonance imaging, or positron emission tomography. Further studies should explore a causative link between the neurodegeneration following TBI and self-injurious behavior.

It has been shown that individuals with a previous TBI have higher rates of nonfatal self-harm, suicide, and all-cause mortality than members of the general population [19]. Koponen and colleagues suggested that TBI could cause decades-long vulnerability to psychiatric disorders such as depressive episodes, delusional disorder, and personality disturbances [20]. Ciurli et al. identified a wide range of neuropsychiatric symptoms in individuals with severe TBI, including apathy (42%), irritability (37%), dysphoria/depressed mood (29%), disinhibition (28%), eating disturbances (27%), and agitation (24%) [21]. Upon examination, the patient in this study denied any suicidal ideations but had a history of psychiatric hospitalizations secondary to suicidal ideation. The increased vulnerability to psychiatric disorders and self-harm in TBI patients may have warranted close neurologic and psychiatric follow-up in the decade prior to this visit.

As previously mentioned, TBIs may lead to complex behavioral changes and psychiatric disorders [2,3,21]. The association between behavioral symptoms and TBIs has long been reported in the literature, with one of the first being the famous case of Phineas Gage. After surviving a penetrating intracranial injury that damaged his frontal lobe, he was reported to have changed from being responsible and socially well-adapted to negligent, irreverent, profane, and unable to take responsibility [22]. A case series by Lane et al. similarly reported post-TBI criminal behavior in four patients with a history of severe TBI with frontal lobe injury [23]. The psychiatric sequelae following TBIs have also been shown to have a correlation with the region of injury. The findings of many studies have been inconsistent, showing associations with damage to the prefrontal cortex and left basal ganglia [24,25]. One study showed that after three months of follow-up, patients with lateral frontal lobe damage showed greater severity of depressive symptoms and apathy as compared to those with medial frontal lobe damage [26]. A cross-sectional study by Schönberger et al. showed imbalances between left vs right frontal and parietal viable brain volumes were related to the development of depression [27]. A study by Thomas et al. reported regional cerebral hypoperfusion to the hippocampus and rostral anterior cingulate cortex in chronic TBI may play a role in self-reported affective symptoms such as anger, anxiety, and depression [28]. However, it is important to note that aside from direct insult to neural tissue, behavioral changes could also be caused by a reaction to the loss of self-esteem due to life-altering injuries or loss of functional status. Furthermore, they can be due to environmental changes, such as changes in caregivers or living situations [2]. Therefore, predicting possible behavioral or psychiatric sequelae requires an understanding of the anatomy damaged in the TBI and alterations to the patient’s quality of life or changes in their environment.

In general, guidelines for intervention on non-suicidal self-injury (NSSI) are clearer for adolescents due to higher incidence rates. Treatment for adolescents is indicated when behavior includes multiple episodes of NSSI or a single episode of NSSI if it is used to cope with extreme distress, is medically serious, or requires medical attention such as sutures [29]. In addition, early intervention in patients with NSSI is critical due to their increased risk for suicidal attempts [30,31]. The patient discussed in this case met indications for intervention. The patient did receive early intervention after the onset of his self-injurious behavior; however, the psychiatry team signed off after initial recommendations were made. It is unknown if closer outpatient follow-up with psychiatry would have stemmed the patient’s habit of biting himself. Additionally, the data published on intervention for NSSI do not explicitly address biting, chewing, or eating one’s own body tissues, as was seen in this case. There have been case reports of individuals specifically biting or chewing on themselves, even eating tissue. However, these reports did not involve patients with a history of TBI [15,16]. Therefore, it is unclear if the etiology of the reason for auto-cannibalism would make a difference in the therapeutic approaches to curtailing the behavior. 

Although there have been suggested algorithms and consensus guidelines for auto-cannibalism, further research is still required to solidify prevention methods and necessary clinical interventions. The current body of evidence does not give sufficient answers as to the best treatment [29]. Given that auto-cannibalism is such an extremely rare disease, more case reports should be published as they arise to gain some insights as to therapeutic approaches. In addition, when dealing with such a disabling disease, gaining accessible information on both failures and success of therapeutic approaches of cases is vital.

## Conclusions

Auto-cannibalism presents not only as a diagnostic but also as a management challenge. A more comprehensive understanding of the factors leading to these self-destructive behaviors secondary to TBIs is needed to improve the outcomes. This case depicts the importance of regular, long-term psychiatric, and neurological follow-up for patients having depressive and anxiety episodes post-TBI, regardless of whether they were previously deemed stable.
